# The role of EBV in the pathogenesis of Burkitt’s Lymphoma: an Italian hospital based survey

**DOI:** 10.1186/1750-9378-9-34

**Published:** 2014-10-15

**Authors:** Giuseppe Pannone, Rosanna Zamparese, Mirella Pace, Maria Carmela Pedicillo, Simona Cagiano, Pasquale Somma, Maria Elena Errico, Vittoria Donofrio, Renato Franco, Annarosaria De Chiara, Gabriella Aquino, Paolo Bucci, Eduardo Bucci, Angela Santoro, Pantaleo Bufo

**Affiliations:** Department of Clinical and Experimental Medicine, Institute of Pathological Anatomy, University of Foggia, Foggia, Italy; Section of Pathological Anatomy Ospedale di Ascoli, Ascoli Piceno, Italy; Section of Pathological Anatomy, Ospedale dei Colli – Monaldi, Napoli, Italy; Section of Pathological Anatomy, Paediatric Oncological Hospital Pausillipon, Naples, Italy; Pathology Unit - Istituto Nazionale dei Tumori, Naples, Italy; Department of Odontostomatological and Maxillofacial Sciences, University of Napoli ‘Federico II’, Naples, Italy; Department of Laboratory, Institute of Histopathology and Diagnostic Cytopathology, Fondazione di Ricerca e Cura ‘Giovanni Paolo II’-UCSC, Campobasso, Italy; Piazza Attilio Omodei Zorini, 48, int.6 00166, Rome, RM Italy; IRCCS CROB - Basilicata Cancer Institute, Rionero in Vulture, Potenza, Italy

**Keywords:** Burkitt’s lymphoma, Immunohistochemistry, In situ hybridization

## Abstract

The exact worldwide incidence of Burkitt’s lymphoma is not known. There are three distinct clinical variants of Burkitt’s lymphoma, each manifesting differences in epidemiology, clinical presentation, morphology, biology and genetic features: the endemic (African), the sporadic (non-endemic), and the immunodeficiency-associated form. In particular, we reported data regarding Burkitt’s lymphoma incidence in the world and across different European countries. Finally, we described clinic-pathological data of 48 Burkitt’s lymphomas occurred in Italy from 2003 to 2013, in 4 different hospitals, two of which located in east side, and the other ones located in the west-coast. Forty Burkitt’s lymphomas occurs in children (age range 3–12), and 8 were adulthood Burkitt’s lymphomas (age range 18–87). In the pediatric group the Male:Female ratio (M:F) was of 4:1, whereas the group of the adult patients has a M:F of 1:1.67. Immunohistochemical detection of Latent Membrane Protein 1 (LMP1) expression and Epstein-Barr virus Encoded RNA (EBER) In Situ Hybridization (ISH) procedures have been performed. Lymphocyte B monoclonal spread has been demonstrated using a Polymerase Chain Reaction (PCR) based method to amplify Fragment Restriction FR1, FR2 and FR3 immunoglobulin heavy chains DNA fragments. Only 38 cases out of 48 were analyzed for LMP-1 showing various percentage of stained cells in 47.4% of the patients.

Considering ISH for EBER detection results:

- 1 out 2 (50%) adult analyzed cases was positive, with 50% of stained tumor cells (this patient was a 22 years old female, coming from Napoli);
- 15 out 24 (62.5%) children analyzed Burkitt’s lymphomas resulted as positive for EBER;
- the overall positivity has been observed in 16/26 Burkitt’s lymphomas (61.53%).
- Finally, EBV has been detected in children and adult patients, one of them with deregulation of the oncogene c-MYC by chromosomal translocation.

## Review of literature

### EBV, Burkitt’s Lymphoma and tumorigenesis

Epstein Barr virus (EBV) or Human Herpesvirus 4 is an important example of a transforming virus belonging to the genus Lymphocryptoviridae, the gamma 1 subtype of the subfamily Gammaherpesviridae and is one of the most common viruses in humans. It is able to infect more than 95% of all individuals within the first four decades of life.

EBV is usually acquired in early childhood in developing countries, with no specific characteristics other than the general symptoms of acute viraemia. However, in developed countries the infection is usually delayed until adolescence or early adulthood years where it is associated with the clinical syndrome referred as infectious mononucleosis. Following primary infection, EBV persists lifelong in the host, selectively infecting in a latent state memory B lymphocytes reservoir. The main viral genes involved in the transformation and persistence of infected B cells are LMP1 and LMP2a. Through self-aggregation on the surface of the infected B cell, LMP1 and LMP2a provide active signals leading to the proliferation of transformed memory B cells that remain in the host for life [1]. LMP1 and other EBV proteins such as Epstein Barr Nuclear Antigen 2 (EBNA2), are highly antigenic, marking the infected B cells for destruction by cytotoxic T cells (CTLs) in the healthy individual. On the other hand, in immunodepressed patients as well as in transplant recipients undergoing immunosuppression, CTLs are immobilized, allowing for the proliferation and immortalization of infected B cells [1]. EBV has been demonstrated to be involved in the development of numerous malignancies, both in immunocompetent hosts and in immunocompromised individuals [2]. The first association was with the endemic Burkitt’s lymphoma. The EBV genome is detected in the majority of neoplastic cells in all patients affected by endemic Burkitt’s lymphoma [3, 4] and there is strong epidemiological association with endemic malaria [5], although there has never been a conclusive population study in support of a direct role of malaria in causation of Burkitt’s lymphoma.

EBV may be detected also in approximately 30% of sporadic Burkitt’s lymphoma cases and it is identified in 25-40% of immunodeficiency-associated Burkitt’s lymphomas [6].

A setting of profound immunosuppression, as in the terminal phases of Human Immunodeficiency Virus (HIV) infection or in organ transplant recipients, leads to loss of control of EBV infection, allowing deregulated proliferation of EBV infected lymphocytes. In this case a spectrum of EBV-driven lymph proliferation, ranging from hyperplasia to frank Non Hodgkin Lymphoma (NHL) could derive [7]. We have to remember that the majority of EBV infections that occur after transplantation, especially in adults, are clinically silent reactivations. This leads to a subsequent delay in the diagnosis of lymph proliferative disorders. Studies have shown a positive correlation between the level of EBV DNAemia after transplantation and the development of post-transplant lymph proliferative disorders (PTLD) which has significant implications in order to monitor and quantify EBV-DNA load after transplantation as a prognostic marker for the development of PTLD [8–10]. Other lymphomas (subtypes of both Hodgkin’s and non-Hodgkin’s lymphomas) are also known to be associated with EBV infection. Epithelial malignancies such as lymphoepitheliomas of nasopharynx [11] and gastric carcinoma [12] are currently included in the list of EBV associated tumours.

In a recent review, Thorley-Lawson et al. [13] described the relationship between EBV infection and Burkitt’s lymphoma. The tumour cells were found to express EBNA in their nuclei, a serologically defined, putative tumour antigen, composed of six components, of which only one, EBNA-1, was expressed in EBV-positive Burkitt’s lymphomas [14]. One observation that favored the carcinogenic role of EBV in Burkitt’s lymphoma was the finding that EBV was an extremely potent transforming virus in culture for the same cell type that develops into Burkitt’s lymphoma, the B lymphocyte [14], being able to convert >50% of B cell into continuously proliferating, latently infected lymphoblastoid cell lines (LCLs) within a few days.

The link between EBV and human cancer is constantly evolving and it lies in the molecular events occurring in Burkitt’s lymphoma pathogenesis.

EBV can display three patterns of latent gene expression: latency I (latency programme), II (default programme) and III (growth programme). Latency III is characterized by expression of all the latent genes (EBNAs, LMPs and EBERs) and occurs on primary infection of B cells. In contrast, persistent infection in vivo is characterized by expression of EBNA-1 and LMP-2 plus the EBERs [15]. In latently infected LCLs, EBV expresses the full spectrum of latent genes; two small nuclear RNAs (EBERs), the highly spliced BamHI rightward transcripts (BARTs), three integral latent membrane proteins, (LMP1, −2A and -2B) and six EBV nuclear antigens (EBNA1, −2, −3A, −3B, −3C, and EBNA-LP) [16].

While EBNA-1 and the EBERs (latency I) have been generally thought to be the only EBV genes expressed in endemic Burkitt’s lymphoma, with a specific role in prevention of apoptosis and survival of neoplastic cells, other recent studies have found that a minor proportion of these tumours has a novel form of latency with a different and broader gene expression profile than previously thought in which the EBNA-3A, 3B, 3C and LP latent genes are expressed in the absence of EBNA-2 and LMP-1 or 2 [17]. Subsequentely, further investigation has demonstrated that endemic Burkitt’s lymphomas may be constituted of tumour cells expressing variable patterns of EBV gene expression, each of which confer a different level of resistance to apoptosis [17]. Thus, EBNA-1, 3A, 3B, 3C and LP positive and EBNA-2, LMP negative Burkitt’s lymphoma cells were the most resistant to apoptosis, while EBNA-2 positive, LMP1-negative Burkitt’s lymphoma cells displayed reduced but “intermediate” resistance [18, 19].

Other studies suggested that biopsies performed on Burkitt’s lymphomas mass can express additional latent proteins, including LMP1, LMP2A and EBNA2 [3]. In particular, LMP2A increases the levels of prosurvival B Cell Lymphoma (Bcl) family members in B lymphocytes, allowing for bypass of p53 inactivation in a MYC tumor model. Recently, some Authors have proposed a role for LMP2A early in development of Burkitt’s lymphoma, where the survival signal allows for expansion of cells that contain a MYC translocation. The expanded cells increase the probability of acquiring a p53 mutation, leading to tumor progression. After the p53 mutation, the tumor cells become less dependent on LMP2A and immune selection may explain the low levels of LMP2A present in tumor biopsies [20].

### Burkitt’s lymphoma

Burkitt’s lymphoma is a highly aggressive B cell NHL with an extremely short doubling time that often presents in extra nodal sites or as an acute leukemia. No single parameter (such as morphology, genetic analysis or immunophenotyping) can be used as the gold standard for the diagnosis of Burkitt’s lymphoma, but a combination of several diagnostic techniques is necessary.

### Epidemiology

The exact worldwide incidence of Burkitt’s lymphoma is not known, as collection of these types of epidemiologic data is limited by a lack of resources that are needed for case ascertainment and accurate diagnosis in the developing countries that have the highest apparent incidence (eg, equatorial Africa) [21].

There are three distinct clinical variants of Burkitt’s lymphoma, each manifesting differences in epidemiology, clinical presentation, morphology, biology and genetic features: the endemic (African), the sporadic (non-endemic), and the immunodeficiency-associated form. The endemic and sporadic clinical variants of Burkitt’s lymphoma differ geographically.

- **Endemic Burkitt’s lymphoma** occurring in Equatorial Africa, Papua New Guinea, represents the most common childhood malignancy of these areas, and shows geographic occurrence corresponding to the geographical distribution of endemic malaria [22]. Burkitt’s lymphoma accounts for 30 to 50 percent of all childhood cancer in equatorial Africa with an estimated incidence of 3 to 6 cases per 100,000 children per year [21]. The peak incidence occurs in children age four to seven years, and the male:female ratio is approximately 2:1.
- **Sporadic Burkitt’s lymphoma** occurring in the US and Western Europe children and young adults. This variant accounts for 30-50% of all childhood lymphomas and less than 1 percent of adult NHLs in the US [23], with an estimated incidence of approximately three cases per million persons per year in both children and adults. In Europe, the incidence is approximately 2.2 cases per million persons per year [24]. The peak incidence occurs in children age 11 years. Among adults, sporadic Burkitt’s lymphoma is typically seen in patients less than 35 years of age, with a median age at diagnosis of 30 years [25]. The majority of patients are males with a 3 or 4:1 male:female ratio [22, 26, 27].
- **Immunodeficiency-associated Burkitt’s lymphoma** occurring in association with the HIV infection, and less commonly in patients with other causes of immunodeficiency (e.g., recipients of organ transplants). In HIV + patients, Burkitt’s lymphoma typically occurs as the initial manifestation of the acquired immunodeficiency syndrome and affects those still immune competent patients with a relatively high CD4 count (e.g., >200 cells/microL) and no opportunistic infections [28], in this way suggesting that HIV itself may have an oncogenic role [29]. In comparison to the majority of other HIV-associated lymphomas, the rate of Burkitt’s lymphoma in the HIV-positive population has not decreased with the advent of Highly Active Anti-viral Therapy (HAART).

The geographic distribution of Burkitt’s lymphoma identifies high, intermediate and low-risk areas [30]. The area of highest risk for Burkitt’s lymphoma appears to be between 10° north and 10° south of the equator and in Papua New Guinea. The zone of intermediate risk for Burkitt’s lymphoma encompasses Southern Europe (Spain, France and Portugal), North Africa, and Asia as far west as Iraq and Kuwait Countries in this zone include also Denmark and the Netherlands since both have slightly elevated Age Standardized Rates compared with the general pattern in Northern and Eastern Europe. The zone of low risk includes most of the remainder of Northern and Eastern Europe, and North and parts of South America and East Asia. In these areas Burkitt’s lymphoma accounts for 6-15% of lymphomas.

### Etiology

Burkitt’s lymphoma was first described in 1958 by a British surgeon working in Uganda who noted a unique, rapidly growing jaw malignancy in children, that was especially common in low altitude, high rainfall areas with a mean temperature over 16 Celsius degrees [31]. The distribution corresponded with that of holoendemic malaria [32], implicating malaria in the aetiology. In 1964, the EBV was identified in cultured cell-lines of the tumour [33], and its consistent presence in African Burkitt’s lymphoma implicated a virus in the aetiology of a human cancer for the first time [34]. The EBV is found in up to 95% of Burkitt’s lymphoma tumours from high-risk areas [35], but in fewer than 30% from low-risk countries [36]. Areas of intermediate risk have intermediate proportions of EBV positive (EBV+) tumours [37]. However, recent works suggest that a low socio-economic status and an early EBV infection can be associated with a higher prevalence of EBV + Burkitt’s lymphoma in low-incidence areas. In immunodeficiency-associated cases EBV is identified in only 25-40% of the cases. However epidemiological studies suggest that malaria and EBV or HIV alone cannot account for the distribution of endemic Burkitt’s lymphoma in high risk countries. Clustering in time and space, and within same families has been observed, particularly in areas of high incidence, but so far these studies have failed to firmly implicate either genetic or environmental factors. Finally, arboviruses and plant tumour promoters are other possible local cofactors.

### Genetics

Different and multiple environmental exposure may converge in a common pathogenetic mechanism involving the *MYC* gene at chromosome 8q24. In fact, all tumours contain the same chromosomal translocations, which culminate in the deregulation of the oncogene c-*MYC*. The translocations involve the *MYC* location (8q24) and one of the immunoglobulin loci on chromosomes 2, 14, or 22 [38, 39]. Most of the cases of Burkitt’s lymphomas presented the *MYC* translocation at band 8q24 to the Immunoglobuline heavy chain locus (IGH) (14q32) or, less commonly, at the lambda (22q11) or kappa (2p12) light chain loci (IGL). The reciprocal translocation t(8:14) occurs in approximately 80% of tumours [40], the remaining 20% being represented by t(2;8) and t(8;22). In African endemic cases, the breakpoint on chromosome 14 involves the heavy-chain joining region and originate from aberrant somatic hypermutation, whereas in sporadic forms, the translocation involves the heavy chain switch region [41]. Finally, up to 10% of the cases may lack a demonstrable *MYC* translocation by Fluorescence In Situ Hybridation (FISH), otherwise evidenced using other molecular techniques. Translocation and deregulation involving *MYC* gene on chromosome 8 is highly characteristic but not specific for Burkitt’s lymphoma. Other genetic and epigenetic alterations can occur in a subgroup of Burkitt’s lymphoma, involving for example TP53 in immune-competent and immune-deficient patients, HIV positive individuals and transplants recipients [42]. In a recent work the first completely sequenced genome from a Burkitt’s lymphoma tumor and germ line DNA from the same affected individual has been described [43]. Authors further sequenced the exomes of 59 Burkitt’s lymphoma tumors, comparing them to sequenced exomes from 94 Diffuse Large B Cell Lymphomas (DLBCLs). 70 genes that were recurrently mutated in Burkitt’s lymphomas, including Inhibitor of DNA binding 3 (ID3), Guanine Nucleotide-binding Protein Alpha 13 (GNA13), Rearranged during Transfection oncogene (RET), Phosphatidyl Inositol 3-Kinase Regulatory Subunit 1 (Pi3KR1) and the Switch/Sucrose Non Fermentable (SWI/SNF) genes, AT Rich Interactive Domain 1A (ARID1A) and SWI/SNF-related Matrix-Associated Actin-Depended Regulator of Chromatin subfamily A-member 4 (SMARCA4) have been identified. In particular ID3 mutations occurred in 34% of Burkitt’s lymphomas and not in DLBCLs.

### Histopathology

Despite chromosomal differences, the “endemic” and “sporadic” forms are indistinguishable morphologically and cytologically [44]. The *classical prototype of Burkitt’s lymphoma* is observed in endemic form and in a high percentage of sporadic cases, particularly in children, but in only a minority of sporadic and immunodeficiency associated adult cases. Neoplastic cells are uniform and small-medium sized with round nuclei, similar or smaller to those of histiocytes, and several or multiple small basophilic paracentrally situated nucleoli. Cytoplasm is deeply basophilic, moderately abundant; it can show slight retraction after formalin fixation and contains lipid vacuoles. Neoplastic cells show a diffuse monotonous pattern of growth, a high mitotic count as well as high apoptotic fraction. Characteristically, there are numerous admixed tingible body macrophages, phagocytosing abundant apoptotic debris and creating a *starry-sky* pattern. Some cases, characterized by a limited stage disease and a good prognosis, may also have a florid granulomatous reaction, causing diagnostic problems in the recognition of the tumour. There are cases in which tumor cells exhibit eccentric nucleus with a single central nucleolus: these cases are referred as *Burkitt’s lymphoma with plasmacytoid differentiation* and can be observed more commonly in immunodeficient patient. Other cases, in the past defined as *atypical Burkitt’s lymphoma/Burkitt like lymphoma*, may show greater nuclear pleomorphism with more prominent nucleoli, but fewer in number. Atypical Burkitt’s lymphomas occur more frequently in many cases of sporadic adult forms.

### Immunophenotype

Burkitt’s lymphoma, regardless of subtype typically expresses monotypic surface IgM with light chain restriction, pan-B-cell antigens, including the CD19, CD20, CD22 and CD79a and co-expresses CD10, CD38, CD43, CD77, Bcl6, and p53, but not CD5, CD23, Bcl2 (or only weakly positive in almost 20% of cases, generally adult patients), CD138 or TdT, thus suggesting follicle centre origin. Burkitt’s lymphoma with plasmacytoid differentiation has in addition monotypic cytoplasmic Ig. The proliferation index is near to 100%.

### Clinical features and prognosis

Staging is performed using the Ann Arbor or, more often, the St Jude/Murphy staging system [45]. Approximately 30% of patients present with limited-stage disease (I or II), while 70% present with widespread disease (stage III or IV). Patients often present with bulky disease, a high tumour burden due to its short doubling time, and with a high risk for spread to the central nervous system (CNS) and bone marrow. A minority of patients with Burkitt’s lymphoma presents with leukemic disease, previously classified as ALL (acute lymphocytic leukaemia), L3 type. In endemic Burkitt’s lymphomas, the jaws and other facial bones are the most frequent sites of clinical presentation. The distal ileum, coecum, omentum, gonads, kidneys, long bones, thyroid, salivary glands and breast may also be affected. In sporadic Burkitt’s lymphomas, jaw tumours are rare, while the majority of case are represented by intra-abdominal masses. In immunodeficiency associated forms, nodal localization is frequent as well as bone marrow involvement. Sporadic and immunodeficiency-associated Burkitt’s lymphomas do not share endemic Burkitt’s lymphomas exquisite sensitivity to chemotherapy, therefore historically the prognosis had been poor, particularly among adults. Although the most important prognostic features have yet to be determined, some clinical factors associated with worse outcome in adults and children include older age, advanced stage, poor performance status, bulky disease, high Lactate Dehydrogenase (LDH), and CNS or marrow involvement, unresectable tumour >10 cm in diameter. Among paediatric patients, a poorer prognosis is associated with age over 15 years [46]. A good prognosis is associated with resectable abdominal disease [44]. High-intensity chemotherapy, sometimes combined with CNS prophylaxis, yields excellent survival in children, both with localized disease and with widespread disease [47]. When similar aggressive chemotherapeutic regimens have been administered to adults, good outcomes have been achieved, with overall survival (OS) rates of 50%–70% [48].

### WHO 2008 Classification: a new entity

The WHO provides an overlap category termed “B cell lymphoma, unclassifiable, with features intermediate between diffuse large B cell lymphoma and Burkitt lymphoma”.

These neoplasms are very aggressive lymphomas that share morphological and genetic features of DLBCL and Burkitt’s lymphoma, but for biological and clinical reasons should be not included in one of the two categories. Morphologically these lymphomas are typically composed of a diffuse proliferation of medium-large sized cells with few admixed small lymphocytes and no stromal fibrosis. A starry-sky pattern, as well as many mitotic figures and apoptotic bodies can be observed, resembling Burkitt’s lymphoma.

There is a marked cellular pleomorphism: in some cases, cells resemble those of Burkitt’s lymphoma but with more variation in nuclear size and cellular contour; other cases are morphologically similar to Burkitt’s lymphoma, but with an atypical immunophenotype or genetic features; other cases share the same immunophenotype of Burkitt’s lymphoma but have an intermediate morphology between Burkitt’s lymphoma an DLBCL; in rare cases, defined ‘blastic or blastoid’, the nuclei are very small, resembling lymphoblastic lymphoma.

The diagnosis of this unclassifiable type of lymphomas should not be made in:

- cases of morphologically typical DLBCL with *MYC* rearrangement;
- typical Burkitt’s lymphomas in which a *MYC* rearrangement cannot be demonstrated;
- atypical Burkitt’s lymphomas with a demonstrable IG-*MYC* rearrangement.

Cases that morphologically resemble Burkitt’s lymphoma and or DLBCL may be placed in this category when:

- the immunophenotype is suggestive of Burkitt’s lymphoma (CD10+, Bcl6+, Bcl2-);
- Bcl2 is moderately-strongly positive (double-hit lymphoma with bot *MYC* an BCL2 translocations);
- Ki67 labelling expression is heterogeneous (50-100%);
- TdT is positive

These intermediate lymphomas express B-cell markers and surface Ig, that in so called double-hit cases can stain negative.

Approximately 35-50% of the cases have 8q24/*MYC* translocations. Many cases have non IG-*MYC* translocations, approximately 15% having a BCL2 translocation, sometimes also together *MYC* translocations (double-hit lymphomas). Less frequently, BCL6 translocation have been observed together *MYC* and/or BCL2. These type of double/triple hit lymphomas reflect a complex karyotype and are more common in elderly patients [25].

### Differential diagnosis

The main diagnostic problem is to differentiate Burkitt’s lymphoma from other types of high-grade B-cell lymphoma, especially from diffuse large B-cell lymphoma. This problem is most evident for adults because a much lower proportion of NHLs in adults is Burkitt’s lymphoma if compared with children population, and because adults (more than children) often have the atypical Burkitt’s lymphoma variant, with morphologic features resembling those of diffuse large B-cell lymphoma.

The immunophenotypic prototype of Burkitt’s lymphoma is IgM+/CD10+/bcl-2–/bcl-6+ with the Ki-67 proliferation index (PI) nearly at 100%; however, cases with an aberrant immunophenotype (such as bcl-2 expression) exist [49]. Occasional diffuse large B-cell lymphomas may exhibit a very high PI, have medium-sized tumor cells showing slight nuclear pleomorphism, with or without a starry-sky pattern (DLBCL-HPSS). Furthermore, some DLBCLs may share immunophenotypic features of Burkitt’s lymphoma [50]. Furthermore, *c-MYC* rearrangement is not unique in Burkitt’s lymphoma and may occur in DLBCLs [49]. These gray-zone cases is an important diagnostic challenge, because the distinction is of great clinical significance for the different treatment strategies for Burkitt’s lymphoma and DLBCL [45]. Generally, we can consider the following key-points to make a correct differential diagnosis.

Features that favour Burkitt’s lymphoma include morphology, an immunophenotype that is CD20+, CD10+, Bcl-6+, Bcl-2−, TdT−, and monotypic sIg+, with virtually all cells Ki67+ (proliferation), and a translocation involving c-Myc and IgH or IgL, without rearrangements involving the bcl-2 or bcl-6 genes [49]. Features that rule out the diagnosis of Burkitt’s lymphoma include BCL6 gene rearrangement, independent from bcl-6 nuclear staining, bcl-2 positivity, presence of t(14;18) and a ki67 staining less than 95%. *c-MYC* protein expression has been suggested to favour Burkitt’s lymphoma over DLBCL, but rare cases of Burkitt’s lymphoma can be c-*MYC* protein negative and some large B-cell lymphomas also express *c-MYC* protein (5%–15% of them having a *MYC* rearrangement) [51].

Studies of micro RNA (miRNA) profiling have evidenced the molecular differences existing between Burkitt’s lymphoma and DLBCL and have demonstrated that the three Burkitt’s lymphoma variants are representatives of the same biological entity with only marginal miRNA expression differences between endemic and sporadic form [52]. In particular, a signature of 38 miRNAs containing *MYC* and nuclear factor-κB (NF-κB) pathway-associated miRNAs has been obtained, differentiating Burkitt’s lymphoma from DLBCL.

Other type of lymphoproliferative diseases (follicular lymphoma, mantle-cell lymphoma, and plasma-cell myeloma) infrequently share *MYC* translocations. In lymphomas other than Burkitt type, c-myc is more likely to have variant translocations (with IgL or other non-Ig partners, rather than IgH), and neoplastic cells tend to have more complex karyotypic abnormalities.

Other entities taking part in the differential diagnosis of Burkitt’s lymphoma include T lymphoblastic lymphoma/leukemia (expressing T cell markers and TdT) and blastoid mantle cell lymphoma (CD5 and cyclin D1 positive).

The florid follicular hyperplasia, with highly active follicle centers with many blast cells and tingible body macrophages, overlaps with the *starry-sky* appearance and with the immunophenotype of Burkitt’s lymphoma. Demonstration of monotypic surface immunoglobulin can definitively exclude a reactive process.

## Clinic-pathological data of Burkitt’s lymphoma cases occurred in Italy from 2003 to 2013. A representative four hospitals based survey

### Patient cohort characteristics

Upon approval by the Ethical Committees of the participants institutions, anatomic pathologists and physicians from four Italian hospitals were asked to furnish data regarding Burkitt’s lymphomas occurred in Italy in the last ten years (2003–20013). Two hospitals were located in east Italy: University Hospital of Foggia and General Hospital of Ascoli Piceno. The other hospitals are located in the west-coast of Italy: General Hospital - AORN Ospedale dei Colli ‘Vincenzo Monaldi’, Napoli) and Children University Hospital - Ospedale Santobono Pausillipon, Napoli. A total of 48 cases of Burkitt’s lymphomas has been recorded. Patients came from Foggia, Napoli and Ascoli Piceno and their broad provinces.

The study population consisted of 40 children and 8 adults. In children group there were 32 boys and 8 girls with a male to female ratio of 4:1; in the adult population there were 3 males and 5 females with a M:F of 1:1,65. Concerning the primary clinical presentation, our Burkitt’s lymphomas of the adults occur as a node mass in 3 cases and as abdominal mass as well as iliac and appendiceal mass in one case respectively. Abdominal mass is the most common manifestation of Burkitt’s lymphoma of the child in our study, this presentation occurring overall in 12 patients (30%). In eight children (20%) it arises as superficial nodes mass in 4 cases (10% of all lymphomas) involving cervical nodes, in 2 as inguinal nodes mass and in 2 as axillary node mass (coinciding with 5% of all lymphomas). In 2 patients the disease arises with tonsils involvement and in other 2 cases with pleural effusion. Furthermore, the primary presentation with liver, retroperitoneum, mesocolon nodes, small bowel involvement and as a pelvic or mesenteric mass regards individuals patients (each case represents the 2,5% of all lymphomas). Staging is performed using the St Jude/Murphy staging system [45]. All patients and/or their relatives gave their informed written consent.

## Materials and methods

The specimens from all cases were fixed in 10% formalin, processed by routine methods, and embedded in paraffin. The final diagnosis of Burkitt’s lymphoma, obtained comparing morphological features with immunohistochemical results for a panel of antibodies including CD3, CD5, CD20, CD10, CD79a, bcl-2, bcl-6 and Ki-67 (MIB-1), was carefully reviewed at the Section of Pathology of the University of Foggia by two pathologists (GP and RZ) and showed in Table 1.

**Table 1:** **Clinico-pathological features of Italian study population and results regarding immunohistochemical findings**

Case	Italian City (Region)	Year	Age	Sex	Site	Immunohistochemistry (IHC)								
						CD20	CD79a	CD10	CD3	CD5	Bcl-6	Bcl-2	Ki67	LMP1
**Case 1**	Foggia (Puglia)	2012	8	M	Tonsil	P	P	P	N	n.d.	P	N	>90	N
**Case 2**	Foggia (Puglia)	2012	52	F	Node	P	P	P	N	N	P	N	>95	N
**Case 3**	Foggia (Puglia)	2006	38	M	Abdominal mass	P	P	n.d.	N	n.d.	n.d.	n.d.	n.d.	n.d.
**Case 4**	Ascoli Piceno (Marche)	2005	41	F	Node	P	n.d.	N	N	n.d.	P	N	100	N
**Case 5**	Ascoli Piceno (Marche)	2006	18	F	Node	P	n.d.	P	N	n.d.	P	P	100	N
**Case 6**	Ascoli Piceno (Marche)	2011	87	M	Ileum	P	n.d.	N	N	n.d.	P	n.d.	98	P
**Case 7**	Ascoli Piceno (Marche)	2012	37	M	Appendix	P	P	n.d.	N	n.d.	P	N	100	n.d.
**Case 8**	Naples* (Campania)	2003	CHILD	M	Retroperitoneum	P	P	n.d.	N	n.d.	n.d.	n.d.	>95	P
**Case 9**	Naples* (Campania)	2003	CHILD	M	Abdominal mass	P	P	n.d.	N	n.d.	n.d.	n.d	>95	P
**Case 10**	Naples* (Campania)	2003	CHILD	M	Small bowel	P	P	n.d.	N	n.d.	n.d.	n.d.	>95	P
**Case 11**	Naples* (Campania)	2003	CHILD	M	Ileum	P	P	n.d.	N	n.d.	n.d.	n.d.	>95	n.d.
**Case 12**	Naples* (Campania)	2003	CHILD	F	Mesocolon nodes	P	n.d.	n.d.	N	n.d.	n.d.	N	>95	P
**Case 13**	Naples* (Campania)	2004	4	M	Abdominal mass	P	P	P	N	n.d	P	N	>90	P
**Case 14**	Naples* (Campania)	2004	5	M	Cervical nodes .	P	P	P	N	n.d	n.d	n.d	>95	n.d.
**Case 15**	Naples* (Campania)	2004	CHILD	M	Axillary nodes	P	P	n.d	N	n.d	n.d	n.d	>95	n.d.
**Case 16**	Naples* (Campania)	2004	CHILD	F	Nasopharinx	P	P	n.d	N	n.d	n.d	n.d	>95	P
**Case 17**	Naples* (Campania)	2005	6	M	Abdominal mass	P	P	P	N	n.d	P	N	>95	P
**Case 18**	Naples* (Campania)	2005	6	M	Inguinal nodes	P	P	P	N	n.d	P	N	>95	n.d.
**Case 19**	Naples* (Campania)	2005	4	F	Pelvic mass	P	P	P	N	n.d	P	N	>95	P
**Case 20**	Naples* (Campania)	2005	3	F	Ileum	P	P	P	N	n.d	P	N	>95	N
**Case 21**	Naples* (Campania)	2005	11	M	Axillay nodes	P	P	P	N	n.d	P	N	100	N
**Case 22**	Naples* (Campania)	2005	4	F	Ileum	P	P	P	N	n.d	P	N	90	P
**Case 23**	Naples* (Campania)	2005	12	M	Abdominal mass	P	P	P	N	n.d	P	N	>95	N
**Case 24**	Naples* (Campania)	2006	5	M	Inguinal nodes	P	P	P	N	n.d	P	N	100	P
**Case 25**	Naples* (Campania)	2006	8	M	Abdominal mass	P	n.d	P	N	n.d	P	N	>90	P
**Case 26**	Naples* (Campania)	2006	12	F	Ileum	P	P	P	N	n.d	P	Focal P	90	N
**Case 27**	Naples* (Campania)	2006	7	M	Mesenteric mass	P	P	P	N	n.d	P	N	>95	P
**Case 28**	Naples* (Campania)	2006	8	M	Liver	P	n.d	P	N	n.d	P	N	100	P
**Case 29**	Naples* (Campania)	2007	6	F	Abdominal mass	P	n.d	n.d	N	n.d	n.d	N	100	n.d.
**Case 30**	Naples* (Campania)	2007	7	M	nasopharynx	P	n.d	P	N	n.d.	P	N	>95	N
**Case 31**	Naples* (Campania)	2007	7	M	Tonsil	P	n.d	P	N	n.d.	P	N	>95	n.d.
**Case 32**	Naples* (Campania)	2007	4	M	Abdominal mass	P	P	P	N	n.d.	P	N	95	P
**Case 33**	Naples* (Campania)	2007	6	M	Cervical nodes	P	P	P	N	n.d.	P	N	95	N
**Case 34**	Naples* (Campania)	2008	10	M	Abdominal mass	P	P	P	N	n.d.	P	N	>90	P
**Case 35**	Naples* (Campania)	2008	7	M	Nasopharynx	P	P	P	N	n.d.	P	N	100	N
**Case 36**	Naples* (Campania)	2008	6	M	Cervical nodes	P	P	P	N	n.d.	P	N	>95	P
**Case 37**	Naples* (Campania)	2008	11	M	Cervical nodes	P	P	P	N	n.d.	P	N	100	N
**Case 38**	Naples* (Campania)	2008	10	M	Abdominal mass	P	P	P	N	N	P	N	>95	N
**Case 39**	Naples* (Campania)	2009	3	M	Abdominal mass	P	P	P	N	N	P	N	>90	N
**Case 40**	Naples* (Campania)	2010	11	M	Abdominal mass	P	P	P	N	N	P	N	>95	N
**Case 41**	Naples* (Campania)	2010	11	M	Abdominal mass	P	P	P	N	n.d.	P	N	100	N
**Case 42**	Naples* (Campania)	2010	5	F	Nasopharynx	P	n.d.	P	N	n.d.	P	N	100	N
**Case 43**	Naples* (Campania)	2011	6	M	Ileum	P	P	P	N	N	P	N	95	P
**Case 44**	Naples* (Campania)	2012	9	M	Ileum	P	P	P	N	N	P	N	>90	N
**Case 45**	Naples* (Campania)	2012	6	M	Pleural effusion	P	P	P	N	n.d.	P faint	N	>90	N
**Case 46**	Naples* (Campania)	2013	3	M	Pleural effusion	P	P	P	N	n.d.	P	N	100	N
**Case 47**	Naples° (Campania)	2003	67	F	n.d.	P	P	n.d.	N	n.d.	n.d.	n.d.	n.d.	n.d.
**Case 48**	Naples° (Campania)	2003	22	F	n.d.	P	P	P	N	N	n.d.	n.d.	n.d.	n.d.

Legend. *, Ospedale Santobono Pausillipon; °, AORN Ospedale dei Colli -‘Vincenzo Monaldi’; P, positive; N, negative; n.d., not determined.

Immunohistochemical detection of LMP1 Expression and EBER In Situ Hybridization Procedures have been performed and evaluated according to standardized guidelines [53].

In our study In Situ Hybridization (ISH) has been performed using *Ventana® EBER ISH iView Blue Plus Kit.* It is performed using a cocktail of EBV encoded small RNA probes. The intended target is the early RNA transcripts of EBV accumulated in the nucleus of EBV-infected cells as evaluated by a blue reaction that is localized to EBV-infected nuclei.

In our study, Lymphocyte B monoclonal spread has been demonstrated using a PCR based method to amplify FR1, FR2, and FR3 immunoglobulins heavy chains DNA fragments according to manufacturer instructions (Invitrogen, Carlsbad, CA, USA).

At first time the slides were assessed either positive or negative for EBV latent infection. The cases assessed as positive were then examined as regard the percentage value of stained cells.

## Results and discussion

After active infection the EBV resides in a latent form with B cells providing the main cellular reservoir [54]. Latent infection by EBV can occur in three forms (latency I, latency II and latency III), each one being marked by a different viral gene expression profile. In fact, not all stages of EBV latency express LMP-1 and questions have been raised about the sensitivity of the immunohistochemistry (IHC) to detect the virus.

Detection of EBV can be also performed by EBER in-situ hybridization (ISH). EBER actually consists of two small EBV latency transcripts of 166 and 172 bases respectively, called EBER-1 and EBER-2 [55]. These transcripts are non-polyadenlylated and therefore not translated into proteins, detectable by IHC. They are naturally amplified and present at high levels in all latency forms of EBV infection, making them ideal targets for ISH, which is widely considered the gold standard for the detection of EBV latent infection in formalin fixed, paraffin embedded tissue (FFPE), being more sensitive than the immunohistochemical evaluation of LMP-1 expression [56].

**Figure 1:**
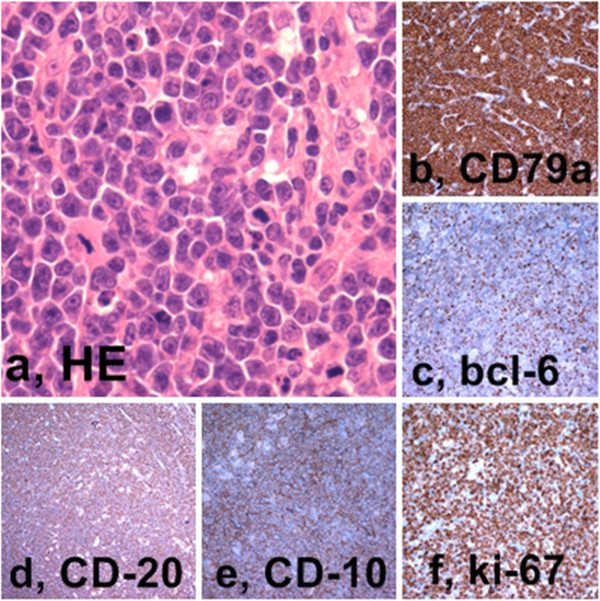
**Burkitt’s lymphoma of the child - Histopathology and immunohistochemistry. a**, Medium sized lymphocytes with high mitotic index and macrophages with tingible bodies (Hematoxylin-Eosin, 60x); **b**, CD79a expression; **c**, bcl6 expression; **d**, CD 20 expression; **e**, CD 10 expression; **f**, Ki 67 expression (LSAB-HRP, nuclear counterstaining with type II Gill’s Haematoxylin).

**Figure 2:**
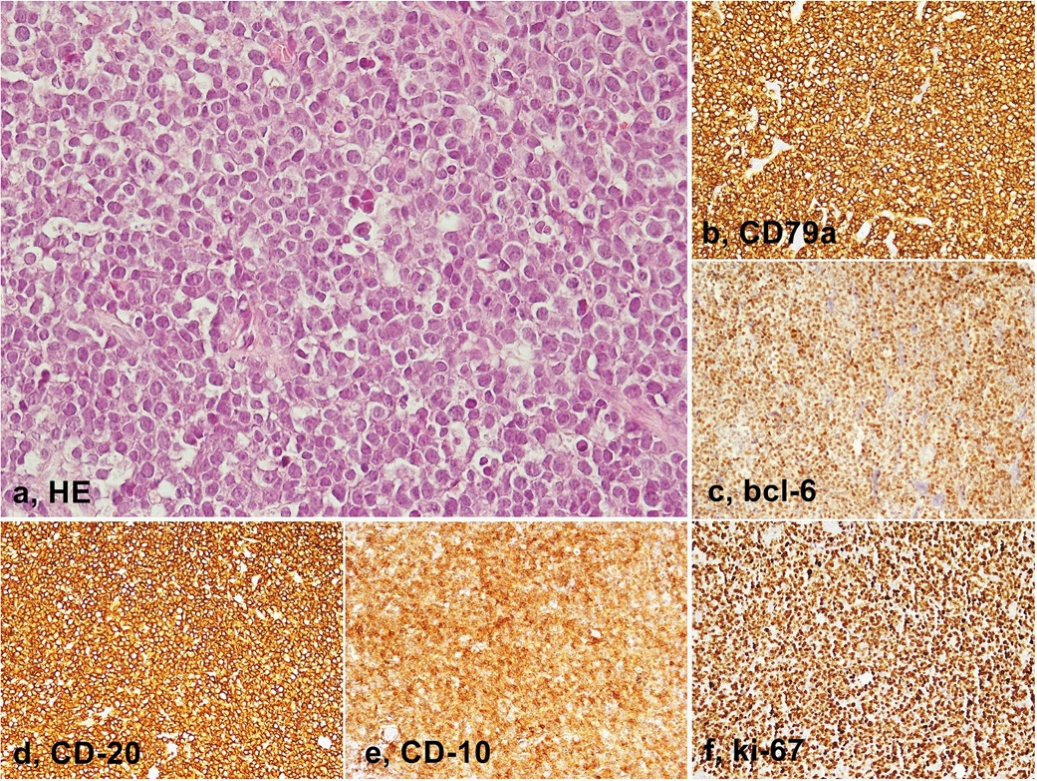
**Burkitt’s lymphoma of the adult - Histopathology and immunohistochemistry. a**, Medium sized lymphocytes with high mitotic index and macrophages with tingible bodies (Hematoxylin-Eosin, 40x); **b**, CD79a expression; **c**, bcl6 expression; **d**, CD 20 expression; **e**, CD 10 expression; **f**, Ki- 67 expression (LSAB-HRP, nuclear counterstaining with type II Gill’s Hematoxylin).

**Figure 3:**
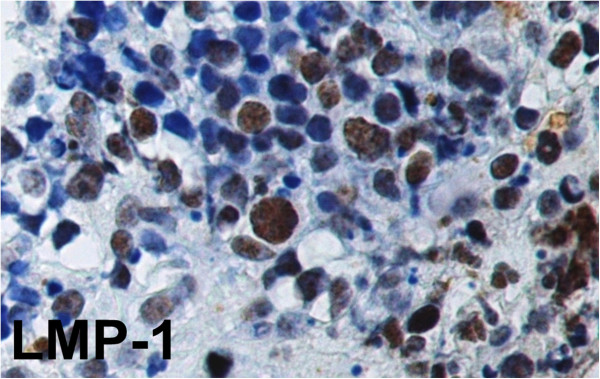
**Immunohistochemical expression of LMP-1 in Burkitt’s lymphoma.** Note the strong nuclear staining of EBV-infected cells. (LSAB-HRP, x400; nuclear counterstaining with type II Gill’s Haematoxylin).

**Figure 4:**
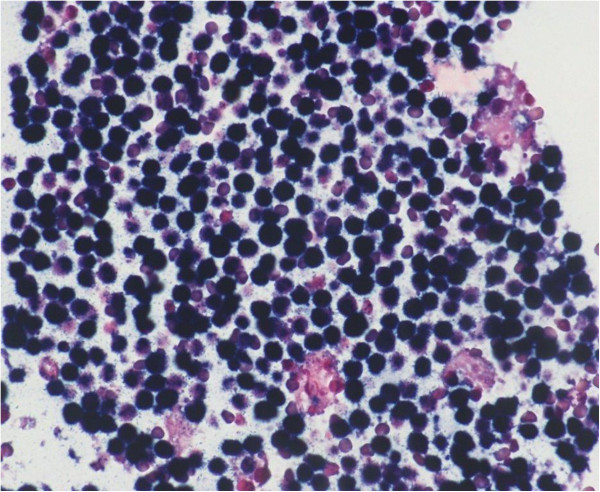
**In situ hybridization for EBER in Burkitt’s lymphoma.** Note diffuse and strong staining showing early RNA transcripts of EBV accumulated in the nucleus of EBV-infected cells (Ventana® EBER ISH iView Blue Plus Kit; original magnification x200).

In our study EBV has been detected both in adult patients (in one of them with deregulation of the oncogene c-MYC by chromosomal translocation) and children by two different diagnostic tests. Histopathologic, immunohistochemical and ISH findings, useful to achieve the final diagnosis of Burkitt’s lymphoma have been reported in Table 1 and showed in Figures 1, 2, 3 and 4.

The immunohistochemical detection of LMP-1 was analyzed in 38 samples (Figure 3).

In the adult population the detection was performed in 4 cases and just 1 expressed LMP-1 (25%). We have information about the presence of deregulation of the oncogene c-MYC by its chromosomal translocation at band 8q24 to the Ig heavy chain region IgH (14q32) in two lymphomas; one of them was precisely the adult Burkitt’s lymphoma positive for LMP-1 (in almost 25% of neoplastic cell). In particular, this case referred to a male patient, 37 years old, living in Ascoli Piceno. He is the only patient without Italian origins, as he comes from the Republic of Peru.

Moreover, one of the studied patients was surely immunodeficient.

In the children subgroup LMP-1 was detected in 34 cases, 17 positively staining for the marker (50%). Overall LMP-1 was positive in 18/38 cases (47.4%) and negative in 20/38 (52.6%). We considered as positive cases with any percent of stained cells. The percentage of stained cells ranged from focal to more than 70% of lymphomatous cells.

Considering ISH for EBER detection results:

- 1 out 2 (50%) adult analyzed cases was positive, with 50% of stained tumor cells (this patient was a 22 years old female, coming from Napoli);
- 15 out 24 (62.5%) children analyzed Burkitt’s lymphomas resulted as positive for EBER;
- the overall positivity has been observed in 16/26 Burkitt’s lymphomas (61.53%).

In children group of Burkitt’s lymphoma, only in 23 cases we have been able to compare IHC results with ISH findings. As diagnostic test, IHC has shown a sensibility of 28.57%, a specificity of 55.5%, a PPV of 50%, a NPV of 33.3% and an accuracy of 39.13%. We have observed a positive concordance IHC-ISH in 17.39% of cases, and a negative one in 21.73%. The total observed concordance was 39.13%, the expected concordance was 10%. The k coefficient was good (0.32).

Our study has demonstrated that although IHC is a good prognostic indicator when used in combination with molecular methods, it is not satisfactory when evaluated as detecting test as used alone. Adding ISH for EBER has the advantage to preserve the morphological context of signals in FFPE samples and increased the sensitivity of diagnostic EBV detection.
